# Treatment of Pulmonary Tuberculosis

**Published:** 1915-07

**Authors:** E. C. Thrash

**Affiliations:** 428 Candler Bldg., Atlanta, Ga.


### TREATMENT OF PULMONARY TUBERCULOSIS.

By E. C. Thrash, M. D.

It would be as impossible to discuss fully this subject in ten minutes as it would be to give the treatment of the whole category of diseases in so short a time, as tuberculosis is so intermingled with the great ocean of human frailties that it touches almost every shore of pathology.

We shall primarily divide the treatment into prophylactic and curative. The prophylaxis of tuberculosis is a problem which is as yet almost wholly unsolved, and there probably is no phase of medical science where there has been so much lost motion as has been in this on account of its having been largely in the hands of unscientific men and hampered by politics. Every case of open tuberculosis should be segregated as long as it is a menace to society and should remain so until no germs are found in the sputum. The patient should be examined from time to time after he returns home and should the germs reappear the same safe-guards should be again observed. This would not only protect society, but be best for the sufferer. This, however, is only ideal as sentiment is a factor that cannot be overcome, but it should be as nearly approached as possible.

The next most important step would be to protect children, as primary infections rarely ever take place after childhood. This is contrary to general opinion, but careful observation has caused me to arrive at this conclusion. Clinical types of tuberculosis in adults result almost universally from latent childhood infections. Every child should be taken from the care of a tuberculosis mother immediately after birth and kept away from others infected. The most common cause of tubercular infections, after nursing and immediate contact by handling children, is that of tramping moist, sputum from the streets into the homes. No case of tuberculosis has developed, in my opinion, from street dust, but moist sputum is carried upon the feet, leaving deposits upon the floors and carpets where children are constantly crawling and playing. They get the virulent germs into their mouths where they easily find entrance into the lymphatic or hemic circulation through some part of the alimentary tract.

Time will not permit going into the proper handling of sputum and close contact with tuberculous people, etc., but it is important to state that close association or intimate contact with tuberculous people for a short time does not produce tuberculosis in adults, as it not only takes long continued exposure but most of them have long since become immunized against the disease. The epitome of prophylaxis is, SEGREGATE THE INFECTIOUS AND CARE FOR THE BABIES, THEN ADULTS WILL CARE FOR THEMSELVES. In consequence of expert knowledge being needed in the control of this disease, every state and important municipality should have a tuberculosis commission untrammelled and with full power to handle such a momentous problem.

In discussing the treatment of tuberculosis pursuant to a cure, we must divide the malady into incipient forms, acute active fulminating types and chronic types, which show a moderate degree of resistance, and then divide its therapy into symptomatic and specific. Most cases of incipient tuberculosis get well without being discovered, and a great majority of those which progress until they are discovered recover when properly handled. However, it is important to state here that but few incipient cases are diagnosed as it requires excellent diagnostic acumen to discover the disease until it is moderately advanced.

There are but few drugs in a general way indicated in early tuberculosis. The treatment is to take them immediately from work, place them at rest, observe all symptoms, keep the emunctory organs in good condition, supply them with nutritious and easily digested foods, but guard against the overworked plan of forced feeding, as it is not the intake that produces results but assimilation. The thick coated tongue, sluggishness of intestines and indicanuria are good indices to overfeeding.

The acute fulminating types should be handled quite similarly to the handling of other acute infections as typhoid fever, pneumonia, acute intestinal diseases, etc. They should be kept in bed, given light, liquid or simi-liquid diet and the temperature kept down by means that would suggest themselves as being indicated in each specific case. There are usually no contraindications to antipyretic drugs unless the infection is so profound that there is myocardial weakness or some pre-existing valvular lesions. One of the most important things to be observed is to guard against giving drugs to get curative results. Since the resisting force of nature is the only thing that in any wise check-mates the ravages of rapidly progressing tubercular processes, so one must have constantly in mind only the very best means that will stimulate the vital forces to the highest degree of efficiency.

There is as much difference in handling chronic progressive tuberculosis, and active acute forms, as there would be in handling a case of chronic rheumatoid arthritis and acute articular rheumatism. In chronic forms the patient's nutrition should stand pre-eminently above every thing else, as diet and assimilation should be observed with the greatest amount of care. Judgment should be used as to the amount of rest to be taken, and temperature should not always govern this. For example, frail, weak, poorly nourished persons with normal temperature often should be kept in bed constantly, while more robust active, well nourished persons with an elevation of temperature in some instances need exercise. Keeping patients in bed for long periods of time even if they run a slight temperature is not always wise; as exercise in these cases often changes a slowly progressing case with a downward tendency to a condition in which the patient will show improvement and a gradual return to a more healthy state.

Aside from drugs used in the treatment of general symptoms, I do not consider that there are but two worth mentioning in bringing about improvement in the lung structure direct. These are mercury and iodine. Creosote is beneficial only in an indirect way. It improves the condition of the intestinal tract by allaying fermentationand putrifaction, thereby improving assimilation. In chronic partially arrested fibroid phthisis, 1-4 to 3-8 grains of mercury daily given with gradually increasing doses of iodine will often produce marked beneficial results. I have recently instituted the introduction of iodine in the form of Lugol's solution intravenously. I have given approximately two hundred injections of this drug, beginning with a minimum dose of 1-2 cubic centimeter and gradually increase with the patient's tolerance. The highest amount which I have as yet given is 3 1-2 cubic centimeters. The patient who has been given this amount has shown a marked improvement and feels that he is practically well. Others have shown less tolerance and in some instances the drug has had to be discontinued. My experience so far prompts me to say that many cases of tuberculosis are improved by this procedure, others are slightly improved and some show no improvement at all. Aside from tuberculin and pneumothorax, however, I can say with full assurance that this procedure has given me better results than any other. Time will not permit me to outline details of administration, though there is a special technique which should be observed.

Only two specifics have stood the test in the treatment of this disease. These are vaccines and pneumothorax. What I mean by a specific is not one that absolutely cures every case of tuberculosis, but one that is applied for the specific purpose of curing the disease and in many instances does do so. Vaccines, a term which should be discarded and antigens used in its stead, have a value that has not received its proper estimate in therapy. Incipient tuberculosis responds most beautifully to the proper administration of tuberculin. The technique of this procedure, however, requires as careful study as would be needed to do the most difficult surgery and only experience with careful observation enables one to master it properly. Chronic cases showing some degree of resistance in many cases are also improved with tuberculin. Many of these where there is a mixed infection will also be benefited by administering autogenous vaccines made from the pyogenic germs which are always associated with the tubercle bacilli in open tuberculosis. The doses should be regulated with the amount of reaction and effect produced upon the patient.

Pneumothorax has its field also in the treatment of this disease, but cases should be properly selected and as a rule there should be only one lung involved. There should be no extensive adhesions to the chest wall, the heart in good condition and no pleuritic effusion. The nitrogen gas is given at gradually lengthening intervals in amounts depending upon the intrathoracic pressure until the lung is well compressed. This compression should be kept up from six months to two years. The principal dangers from this procedure are gas emboli in the circulation, gas oedema in the tissue, pressure upon a weakened heart, producing heart failure, and pain in pulling upon pleuritic bands of adhesions.

428 Candler Bldg., Atlanta, Ga.

